# Willingness to pay for new medicines: a step towards narrowing the gap between NICE and IQWiG

**DOI:** 10.1186/s12913-020-5050-9

**Published:** 2020-04-22

**Authors:** Afschin Gandjour

**Affiliations:** Frankfurt School of Finance and Management, Adickesallee 32-34, 60322 Frankfurt am Main, Germany

**Keywords:** NICE, IQWiG, Cost-effectiveness, Threshold

## Abstract

**Background:**

The question of how to set the cost-effectiveness threshold for new, innovative medicines is a matter of ongoing controversy. One prominent proposal suggests that the cost-effectiveness threshold adopted by the U.K. National Institute for Health and Care Excellence (NICE) should represent the opportunity cost for the U.K. National Health Service resulting from the adoption of new medicines. The purpose of this article is to compare this proposal for the U.K. with the approach chosen by the Institute for Quality and Efficiency in Health Care (IQWiG) in Germany, which relies on indication-specific cost-effectiveness thresholds.

**Main text:**

The ‘ideal’ NICE and IQWiG surprisingly share the fundamental principle of inferring the willingness to pay from existing care. For this and other reasons, indication-specific thresholds based on IQWiG’s methodology do not lead to more inefficiency at the health system’s level than a generic threshold based on the ‘ideal’ NICE (keeping other conditions the same). Also, applying either decision rule to one country will yield a similar long-term growth in population spending. Assuming that everything else is equal, both decision rules are predicted to decrease long-term expenditure growth. Convergence of the two decision rules would require, among others, ruling out waste in the ‘ideal’ NICE’s approach and, for IQWiG’s approach, using the same relative weights for life expectancy and health-related quality of life as the quality-adjusted-life-year model.

**Conclusion:**

This article shows that both decision rules have notable commonalities in terms of inferring the willingness to pay from existing care and the projected impact on long-term growth in population spending.

## Background

In the industrialized world there has been a trend towards value-based pricing (VBP) of new, innovative medicines (new therapeutic entities) [1]. According to an Organization for Economic Co-operation and Development paper [2], VBP refers to regulation of reimbursement or pricing of pharmaceuticals on the basis of their therapeutic value. VBP has been suggested both as a way to control health expenditures and to maximize health benefits based on the available resources [3]. VBP defined in a narrow sense uses a threshold incremental cost-effectiveness ratio (ICER, the ratio of additional costs to additional health benefits) for reimbursing or pricing new drugs [4]. The U.K. National Institute for Health and Care Excellence (NICE) currently uses a cost-effectiveness threshold in the range of £20,000 to £30,000 per quality-adjusted life year (QALY) for reimbursing new drugs in the National Health Service (NHS) [5]. Claxton et al. criticize this threshold range for “having little or no empirical foundation” [6]. Instead, they propose setting the maximum willingness to pay for a health care program equal to the cost-effectiveness ratio of health care programs currently funded (or the cost per expected “average of displaced QALYs”) [6]. They argue that under the NHS budget constraint „[a]pplying any threshold that is higher than one that reflects the health that is expected to be displaced will necessary reduce overall health outcomes” [6]. The new threshold thus should reflect the health of unidentified NHS patients who bear the real opportunity costs. One may assume, in line with the authors’ empirical work in assessing the threshold [7], that it is rather marginal care which is displaced under the NHS budget constraint. In this case, the threshold corresponds to the “marginal productivity” [8] or the marginal cost-effectiveness ratio of health care and may change with the budget impact of the new drug [9]. Yet, one may also question this assumption [10], (p. 24/25) and presume that services are displaced at random [11], in line with an ‘uninformative’ prior or an ‘equiprobability’ assumption assigning each service an equal probability of being displaced. In this case, the threshold should reflect the average cost-effectiveness ratio of services provided [11]. Notwithstanding this controversy, the proposal for setting NICE’s threshold is receiving considerable attention and has been considered the favorable approach for resource allocation under a budget constraint [12]. The approach has also been adopted to estimate an ICER threshold for the health care systems of Spain [13] and Australia [14].

**Fig. 1:**
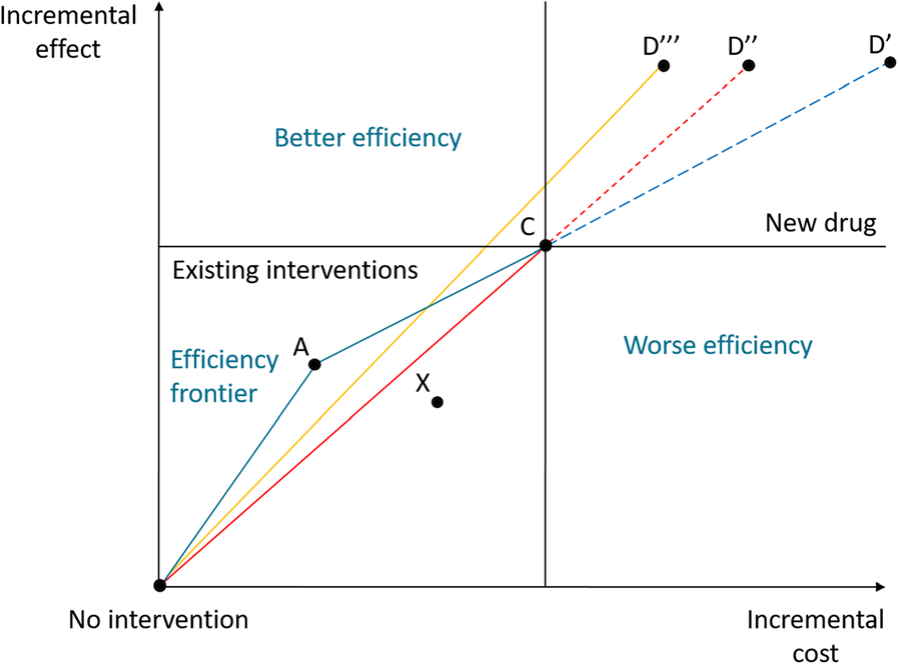
Decision rules for setting reimbursement prices by the Institute for Quality and Efficiency in Health Care (IQWiG). A, C, and X are pairs of incremental costs and effects of existing interventions and D’, D”, and D”’ are cost and effect pairs of the new drug. The reference point is ‘no intervention’

In Germany, the Institute for Quality and Efficiency in Health Care (IQWiG) published its most recent update of its methods for evaluating the relation between costs and benefits in setting reimbursement prices in 2017 [15]. The first official version of the method was published in 2009 [16]. To provide information on reimbursement prices, IQWiG uses the following decision rule^1^ (also called proportional rule [17, 18]): The ICER of a new drug compared to the next effective intervention should not be higher than the ICER of the next effective intervention compared to its next effective alternative. Hence, in order to apply IQWiG’s rule a new drug needs to have two comparators. Two comparators always exist (except for the rare circumstance where they are dominated by the new medicine): one is doing nothing and the other is palliative or supportive care. According to IQWiG, the various alternatives are positioned on a cost-benefit plane (Fig. 1), an “efficiency frontier” (EF) is drawn along non-dominated alternatives (A and C in the figure), and the reimbursement price D’ is determined by an extrapolation of the last segment of the EF (from A to C). A previous methods paper [16] also presents stricter variations of this rule, leading to lower reimbursement prices. They determine reimbursement prices either based on i) the ICER of the currently most effective intervention compared to no intervention [16], thus yielding D” in Fig. 1 or ii) the average cost-effectiveness ratio of all non-dominated alternatives in a therapeutic area [16], thus yielding D”’. In the following we will call IQWiG’s base-case rule ‘marginal rule’ and the rule based on ii) ‘average rule’.

According to IQWiG’s methodology, prices in each therapeutic area are assessed separately, i.e., no direct comparison between therapeutic areas is conducted [15, 19]. Although measures of health benefits may differ between therapeutic areas, they need to be the same for the interventions compared in order to allow establishing the reimbursement price [15, 19]. As potential measures of health benefit, IQWiG allows the use of patient-relevant outcomes such as mortality, health-related quality of life (QoL), morbidity (symptoms and complications), and side effects; validated surrogates of patient-relevant outcomes; and transformations of patient-relevant outcomes into approximately cardinally scaled measures [15]. While IQWiG does not explicitly exclude QALYs as a measure of health benefit, it criticizes their use based on ethical and methodological grounds [15]. As an alternative, IQWiG allows for the use of the analytic hierarchy process and the conjoint analysis to weight the different patient-relevant outcomes [15]. If a new drug has several indications, prices are first set by therapeutic area and may then be aggregated by applying weights [15]. The approach of explicit pricing in each indication (before aggregation) may lend itself towards indication-based pricing.^2^

Despite the fact that IQWiG’s rule neither starts from a budget constraint nor explicitly considers opportunity costs, the notion of inferring a threshold based on the cost-effectiveness ratio of existing health care services is thus also common to IQWiG. That is, the ‘ideal’ NICE (as envisaged in [6] but not necessarily the ‘real’ NICE) and IQWiG surprisingly share the fundamental principle of inferring the willingness to pay from existing care. The similarity is obvious both for IQWiG’s marginal and average rule. For IQWiG’s marginal rule, which derives the willingness to pay based on the marginal cost-effectiveness ratio of all non-dominated alternatives (the last segment of the EF), it is the similarity with the proposal for NICE using the cost-effectiveness ratio of marginal care to define opportunity costs. And for IQWiG’s average rule, which is based on the average cost-effectiveness ratio of all non-dominated alternatives, it is the similarity with an opportunity-cost definition based on the cost-effectiveness ratio of average care. Yet, critics would point out that according to IQWiG’s rule each therapeutic area has its own willingness-to-pay threshold and that no direct comparisons between therapeutic areas are conducted. This is unlike the ‘ideal’ NICE whose explicit goal is to make comparisons across therapeutic areas.

The purpose of this article is to elaborate on the commonalities and differences between the decision rules of the ‘ideal’ NICE and IQWiG. As a word of caution, IQWiG has completed only one cost-effectiveness analysis (CEA) so far [20]. While the German Act on the Reform of the Market for Medicinal Products (AMNOG), which went into effect on 1 January 2011, allows commissioning IQWiG with the conduct of a CEA, so far payers and manufacturers have relied on the compulsory price negotiation to determine prices of new, innovative medicines within the German statutory healthcare system (unless medicines have been assigned to a therapeutic reference group). Information on actual ICER threshold levels in the various therapeutic areas is therefore limited and may only be cautiously derived from CEAs that have been conducted for other purposes. For example, in patients with heart failure the ICER threshold for a new, innovative product is estimated at €23,401 per life-year gained [21], assuming that sacubitril/valsartan and enalapril would be appropriate comparators and that the CEA comparing sacubitril/valsartan to enalapril [21] would fulfil IQWiG’s requirements.

Next to the desire for analytical clarity, this article is motivated by a recent European Commission (EC) proposal on health technology assessment (HTA) [22] to promote convergence in HTA tools, procedures and methodologies. The underlying problems are an “impeded and distorted market access”, which results from different national HTA processes and methodologies, as well as duplication of work for national HTA bodies that needs to be carried out on the same health technologies. While the EC proposal makes a distinction between clinical and non-clinical assessments (e.g. economic, organizational, and ethical) and states that non-clinical assessments would remain at the Member State-level because they are more linked to national contexts (but may be subject to “voluntary cooperation” between Member States), the purpose of this article is to search for alignment in carrying out the (non-clinical) assessments for pricing and reimbursement.

## Main text

### Analytic approach

As stated in the introduction, the purpose of this article is to elaborate on the commonalities and differences between the decision rules of the ‘ideal’ NICE and IQWiG. To this end, the analysis holds a long-term perspective, in line with recommendations for economic evaluations (e.g., [23]). In this theoretical exercise, the decision rules by NICE and IQWIQ are applied to a single country or health system. Hence, the primary intention is to present a comparison between the two decision rules and not between the two countries, the U.K. and Germany. That is, the exercise does not specifically refer to the U.K. or Germany as countries but rather to their decision rules independent to where they are applied. Assuming that everything else is equal (the so-called ceteris paribus assumption), the comparison between the decision rules is neither influenced by contextual factors such as the scope and history of health technology assessment in the two countries nor the validity of the cost-effectiveness estimates (i.e., how well the decision rules are implemented). This is not an outrageous assumption but necessary to isolate the impact of the decision rules and avoid confounding by factors that occur in the real world.

### IQWiG’s rule

According to IQWiG’s decision rule, the threshold ICER *R* is based on the ICER of the comparator (i.e., the most effective treatment in IQWiG’s most recent methods paper [15] or the average treatment in a past version [16], each compared to its comparator, e.g., no treatment). For a new drug adopted in period *i* = 0, *R* can thus be formalized as follows [24]:

1 $$ R=\frac{\Delta {c}_0}{\Delta {e}_0} $$

where Δc and Δe are the incremental costs and effects of the comparator treatment, respectively. As implied by IQWiG’s rule, for new drugs adopted in period *i* = 1 to *n* the threshold ICER stays the same [24]:

2 $$ R=\frac{\Delta {c}_0}{\Delta {e}_0}=\frac{\Delta {c}_1}{\Delta {e}_1}=\frac{\Delta {c}_2}{\Delta {e}_2}=...=\frac{\Delta {c}_n}{\Delta {e}_n} $$

Growth of incremental costs in period *n* can thus be calculated as follows [24]:

3 $$ \frac{\Delta {c}_n}{\sum \limits_{i=0}^{n-1}\Delta {c}_i} $$

Assuming that in each period health gain will be the same (in line with past survival gains, e.g., in the oncology space [25]) and that manufacturers set prices at maximum reimbursement levels (see the Discussion for a relaxation of these assumptions), incremental costs will stay the same too based on the proportional relationship between costs and health gains. That is, we can describe incremental costs in each period by a constant *k* [24]:

4 $$ \Delta {c}_i=k $$

Then, expenditure growth will decrease from period *i -* 1 to the next period *i* [24]:

5 $$ \frac{k}{\left(i-2\right)\cdot k}>\frac{k}{\left(i-1\right)\cdot k} $$

Using Eq. (2) as a basis, we vary *R* by factor *a*: [0,∞) in order to analyze the impact of the level of *R* on expenditure growth:

6 $$ a\cdot R=\frac{a\cdot \Delta {c}_0}{\Delta {e}_0}=\frac{a\cdot \Delta {c}_1}{\Delta {e}_1}=\frac{a\cdot \Delta {c}_2}{\Delta {e}_2}=...=\frac{a\cdot \Delta {c}_n}{\Delta {e}_n} $$

Assuming constant health gains over time, expenditure growth in period *i* is calculated as follows:

7 $$ \frac{a\cdot k}{\left(i-1\right)\cdot a\cdot k} $$

Given that factor *a* cancels out, the threshold value does not have an impact on the growth rate. Hence, whether we extrapolate the cost-effectiveness ratio of the currently most effective intervention (IQWiG’s marginal rule) or the average cost-effectiveness ratio does not matter in terms of expenditure growth. Next, consider that the effects of IQWiG’s rule on expenditures at the system’s level in periods *i -* 1 and *i* can be obtained by aggregating relative threshold values *a*’s specific to single therapeutic areas. Formally:

8 $$ \frac{\sum \limits_{\mathrm{j}=1}^{\mathrm{m}}{a}_{\mathrm{j}}\cdot {n}_{\mathrm{j}}\cdot {k}_{\mathrm{j}}}{\left(i-2\right)\cdot \sum \limits_{\mathrm{j}=1}^{\mathrm{m}}{a}_{\mathrm{j}}\cdot {n}_{\mathrm{j}}\cdot {k}_{\mathrm{j}}}>\frac{\sum \limits_{\mathrm{j}=1}^{\mathrm{m}}{a}_{\mathrm{j}}\cdot {n}_{\mathrm{j}}\cdot {k}_{\mathrm{j}}}{\left(i-1\right)\cdot \sum \limits_{\mathrm{j}=1}^{\mathrm{m}}{a}_{\mathrm{j}}\cdot {n}_{\mathrm{j}}\cdot {k}_{\mathrm{j}}} $$

where *j* refers to a therapeutic area and *n* denotes the number of treated patients. If *n* and *k* stay constant over time (as is assumed by Eq. (8)), the sum terms cancel out and aggregation does not have an impact on expenditure growth. Hence, at the system’s level IQWiG’s rule still leads to a decrease in expenditure growth (regardless of whether it is based on marginal or average care).

Finally, the threshold ICER at the system’s level *R* in period *i* is calculated as a weighted-average of the threshold values in the therapeutic areas (*R*_*j*_). Formally:

9 $$ R=\frac{\sum \limits_{\mathrm{j}=1}^{\mathrm{m}}{R}_{\mathrm{j}}\cdot \Delta {e}_{\mathrm{j}}\cdot {n}_{\mathrm{j}}}{\sum \limits_{\mathrm{j}=1}^{\mathrm{m}}\Delta {e}_{\mathrm{j}}\cdot {n}_{\mathrm{j}}} $$

where ∆e_*j*_ represents added benefits in therapeutic area *j*. As a calculation example, assume a simple case of two therapeutic areas with threshold values (*R*_*j*_) of €20,000 and €40,000 per unit of health benefit, added benefits (∆e_*j*_) of 6 and 12 months, as well as population sizes (*n*_*j*_) of 10,000 and 20,000, respectively. Plugging the factors into Eq. (9), we obtain a threshold ICER at the system’s level of €36,000.

### Comparison to the ‘ideal’ NICE

Now, we turn to the comparison against the decision rule for the ‘ideal’ NICE, which is based on a budget constraint of the NHS. Yet, despite the budget constraint NHS expenditures have grown considerably over time and for the period between 1994 and 2010 even at a higher rate than expenditures of the German statutory health insurance (SHI).^3^ The threshold value of the ‘ideal’ NICE, i.e., the marginal or average cost-effectiveness ratio of existing care depending on the type of services assumed to be displaced, can be formally represented as a weighted average of the marginal or average cost-effectiveness ratios of different therapeutic areas where weights are a product of number of treated patients and size of health gains in the therapeutic areas. Thus, Eq. (9) also applies to the decision rule for the ‘ideal’ NICE. That is, while threshold values in the various therapeutic areas can be aggregated to an average (as in the case of IQWiG) the opposite also holds, i.e., an aggregated average threshold value (such as that of the ‘ideal’ NICE) can be formally decomposed into cost-effectiveness ratios of the various therapeutic areas. This is important to stress because having indication-specific threshold values (as in the case of IQWiG) does not result by itself in more inefficiency (i.e., less aggregated health) at a system’s level.

As a further similarity to IQWiG, the decision rule for the ‘ideal’ NICE (regardless of whether it is based on the average or marginal cost-effectiveness ratio) is also influenced by prices in therapeutic areas that have shown little innovation over the past years. That is, generic prices in such therapeutic areas tend to bring down the cost-effectiveness ratio of the NHS and the threshold ICER. Furthermore, as was also stated for IQWiG’s approach [28], the ‘ideal’ NICE does not preclude interventions that have a high ICER but are still undominated. Moreover, IQWiG’s approach, if applied under a budget constraint, would also displace ‘average’ or ‘marginal’ care (depending on the assumption about the types of services being displaced). Only the perspective is different: In the case of IQWiG displaced services are a consequence of applying the decision rule while in the case of the ‘ideal’ NICE they are causal in determining the decision rule.

On the other hand, it is important to note that average or marginal care as considered by the ‘ideal’ NICE is affected by waste (overuse) in health care [7], i.e., includes dominated alternatives. The latter then have an impact on the willingness-to-pay threshold set by the ‘ideal’ NICE but not on the threshold set by IQWiG, which explicitly excludes dominated alternatives. This results, ceteris paribus, in a higher threshold of the ‘ideal’ NICE and effectively translates into a ‘reward’ for waste in health care.

As a further difference, average or marginal care as considered by the ‘ideal’ NICE does not exclude any type of health care service. Yet, according to IQWiG’s analysis, strictly speaking, only comparators of drugs and their comparators are taken into consideration.

### Quantifying the differences

There is only limited data that allows estimating how the cost-effectiveness of current health care (the threshold for the ‘ideal’ NICE) is impacted by waste. Estimates on waste in international health care systems run in the range of 20% to a third of total health care spending [29]. This means that a threshold ICER that includes waste increases by 25 to 50% compared to a threshold without such consideration.

Also, the cost-effectiveness ratio of medicines (representing the typical comparators of IQWiG’s decision rule) has shown to be somewhat lower than that of other types of health care services [30]. Data from the Tufts Medical Center CEA Registry yielded a median cost-per-QALY ratio of US-$7094 for medicines compared to US-$9041 for all interventions [30]. As a word of caution, the majority of studies included in the Tufts CEA Registry was conducted in the United States. Taking these data at face value raises the threshold ICER of the ‘ideal’ NICE by another 20 to 30% compared to that of IQWiG at the system’s level.

In addition, one may ask whether using clinical endpoints (in the case of IQWiG) as opposed to the QALY measure (in the case of NICE) has implications for the threshold value. Based on the fact that endpoints considered to be patient-relevant from IQWiG’s perspective include both mortality and health-related QoL and thus the components of a QALY, it is not evident a priori whether health benefits are over- or underappreciated compared to the QALY measure. If IQWiG, let’s say, underappreciated/underweighted health benefits from QoL improvement due to missing values, the ICER of comparators (i.e., the threshold ICER) would increase and vice versa.

## Discussion

This article starts from the observation that the ‘ideal’ NICE as envisaged in [6] and IQWiG surprisingly share the fundamental principle of inferring the willingness to pay from existing care. It shows that, for the purpose of pricing or reimbursing new drugs, indication-specific thresholds based on IQWiG’s methodology, ceteris paribus, do not lead to more inefficiency (i.e., less aggregated health) at the health system’s level than a generic threshold based on the ‘ideal’ NICE. Also, applying either decision rule to one country will yield a similar impact on long-term population health expenditure growth. Ceteris paribus, both decision rules are predicted to decrease population health expenditure growth. While relaxing the assumptions of a constant health gain and pricing up to the threshold can lead, respectively, to a growth increase and decrease, both rules are affected to the same degree.

As a word of caution, as the analysis adjusts for contextual factors, real-world implementation of the decision rules in the two countries can lead to divergent expenditure paths, e.g., due to differences in the scope and history of health technology assessment. Moreover, using IQWiG’s decision rule requires a one-time adjustment of health expenditures because, in contrast to the ‘ideal’ NICE, dominated alternatives are excluded and comparators are mainly restricted to medicines. It is this one-time adjustment which, ceteris paribus, leads to a lower threshold value of IQWiG’s rules compared to the rule of the ‘ideal’ NICE if applied to one country (without impact on long-term expenditure growth though as stated).

Hence, while the aggregated impact of the two decision rules differs in the short-term but is similar in the long run, what is the remaining difference between the two decision rules? The impact of the decision rules differs by therapeutic area, and this matters particularly for manufacturers. That is, while the ‘ideal’ NICE implicitly pools cost-effectiveness thresholds pertaining to each therapeutic area, thus yielding one basic threshold value across all therapeutic areas, IQWiG does not allow for pooling and uses a different threshold in each therapeutic area. IQWiG thus provides stronger rewards and incentives for manufacturers in high-cost high-burden therapeutic areas [18, 19] (e.g., cancer care) but lower rewards and incentives in low-cost low burden areas. In contrast, by using an average, the ‘ideal’ NICE reduces rewards and incentives in high-cost high-burden therapeutic areas and increases them in low-cost low burden therapeutic areas. Yet, consideration of the burden of illness is well in line with the VBP framework for branded medicines announced by the U.K. Department of Health [31]. Further research is needed to compare IQWiG’s implicit consideration of burden of illness with the explicit requirements by the U.K. Department of Health.

In summary, the recent proposal for setting NICE’s threshold reduces differences compared to IQWiG’s decision rule. Still, a complete convergence in terms of costs and benefits at the system’s level, when applying either decision rule to one system, would require the following adjustments as summarized from above: i) the threshold for the ‘ideal’ NICE would need to rule out waste; ii) the threshold ICER of the ‘ideal’ NICE would need to be based on the comparators used for the EF method; and iii) the EF method would need to apply the same relative weights for life expectancy and health-related quality of life as QALYs do. On a practical level, the cost-effectiveness calculations underlying the ‘ideal’ NICE and the EF would need to reflect real-world conditions to the same degree.

## Conclusions

This article shows that both decision rules have notable commonalities in terms of inferring the willingness to pay from existing care and the projected impact on long-term growth in population spending. Hence, countries inside and outside Europe implementing an opportunity-cost approach would move in the direction of IQWiG’s EF method. The ‘common denominator’ of both decision rules as described in this paper could be a starting point for further development. At the same time, the differences between the two rules as outlined above can provide a basis for a future research agenda investigating what each country’s decision rule should incorporate and what it should not.

## Data Availability

Not applicable.

## Abbreviations

AMNOG: Act on the Reform of the Market for Medicinal Products
CEA: Cost-effectiveness analysis
EC: European Commission
EF: Efficiency frontier
HTA: Health technology assessment
ICER: Incremental cost-effectiveness ratio
IQWiG: Institute for Quality and Efficiency in Health Care
NHS: National Health Service
NICE: National Institute for Health and Care Excellence
QALY: Quality-adjusted life year
QoL: Health-related quality of life
SHI: Statutory health insurance
VBP: Value-based pricing

## Footnotes

1 The term ‘rule’ is used in its usual sense, i.e., it does not preclude that other criteria may lead to a change in recommendation.

2 This point was made by a reviewer.

3 Real NHS expenditures (i.e., adjusted for inflation) increased by 112% [26], while real SHI expenditures only increased by 18% in the same time period [27].
